# Characteristics of irreversibility in high-resolution computed tomography in chronic hypersensitivity pneumonitis 

**DOI:** 10.5414/ALX02560E

**Published:** 2025-07-02

**Authors:** Beate Rehbock, Andreas Gschwendtner, Okka W. Hamer

**Affiliations:** 1 Institute for Lung Imaging and Image Assessment, Potsdam,; 2 Institute of Pathology, Kulmbach Hospital, Kulmbach, and; 3 Department of Radiology, University Medical Center Regensburg and Caritas St. Maria Lung Clinic, Donaustauf, Germany

**Keywords:** chronic hypersensitivity pneumonitis, high-resolution computed tomography, fibrosis, ground-glass opacity, air trapping

## Abstract

From a radiological point of view, the statement of irreversibility can only be made with certainty for the signs of irregular reticulation in conjunction with traction bronchiectasis and for honeycomb cysts in the HR-CT morphologically fibrotic phenotype. The HR-CT signs of the inflammatory phenotype can behave differently. Ground-glass nodules always have a reversible potential. In contrast, the underlying pathology of ground-glass opacity can only be assessed over time, as it does not necessarily correlate with inflammation but can also correspond to fine fibrosis. Similarly, air trapping on HR-CT is an important sign of hypersensitivity pneumonitis in both the radiologically inflammatory and fibrotic form. The persistence of air trapping in the inflammatory type over years suggests the possibility of irreversibility of the bronchiolitis in these cases. Whether a higher resolution in HR-CT, such as through photon-counting CT, can lead to clearer differentiation and prediction of reversibility and irreversibility in the future remains to be seen.

## Introduction 

The ATS/JRS/ALAT guideline for the diagnosis of hypersensitivity pneumonitis (HP) from 2020 no longer classifies HP according to its temporal course but rather based on its morphology into a non-fibrotic and a fibrotic type [1]. In contrast, the current German S2K guideline on HP (also known as extrinsic allergic alveolitis (EAA)) from 2024 returns to the classification prior to 2020, categorizing HP primarily based on whether an “acute” or “chronic” disease process is present. Only thereafter does it further differentiate whether the chronic course is associated with signs of fibrosis or not [2]. 

High-resolution computed tomography (HR-CT) remains the imaging method of choice for both guidelines in diagnosing HP. Since the disease can affect two anatomical components – the parenchyma and the airways – in a temporally and spatially disseminated manner, the HR-CT findings of HP are diverse. Therefore, the 2020 international guideline established diagnostic criteria for CT imaging for the first time. Similar to the pattern of usual interstitial pneumonitis in the guideline on idiopathic pulmonary fibrosis (IPF), graded probabilities were defined separately for non-fibrotic and fibrotic forms of HP in HR-CT [1]. 

To capture all HR-CT variations of HP, particularly of HP with fibrosis, a very detailed tabular framework was developed, which, due to its complexity, has hardly been applied in routine radiological diagnostics in Germany according to our experience. Thus, from a prognostic-therapeutic perspective, the new German S2K guideline suggests a pragmatic differentiation in HR-CT “only” between an inflammatory and a fibrotic phenotype [2]. 

## HR-CT signs and distribution in HP 

The inflammatory type is predominantly characterized on HR-CT by signs associated with ground-glass opacities (diffuse ground-glass opacities, ground-glass nodules, 3-density sign). Additionally, other signs of airway disease, such as air trapping and cysts, may be detectable. The distribution is usually bilateral and diffuse or multilocular. 

To classify as fibrotic HP, manifest signs of fibrosis like traction bronchiectasis and/or honeycomb cysts are required. Variably, signs from the inflammatory type and/or phenomena of airway disease like air trapping may also be present. In the fibrotic phenotype of HP, there are cases that show a combination of fibrosis with inflammatory signs on HR-CT, as well as “pure” fibrotic HP cases. The distribution can vary significantly in both cranio-caudal and axial gradients. 

## Reversibility versus irreversibility 

**Figure 1:**
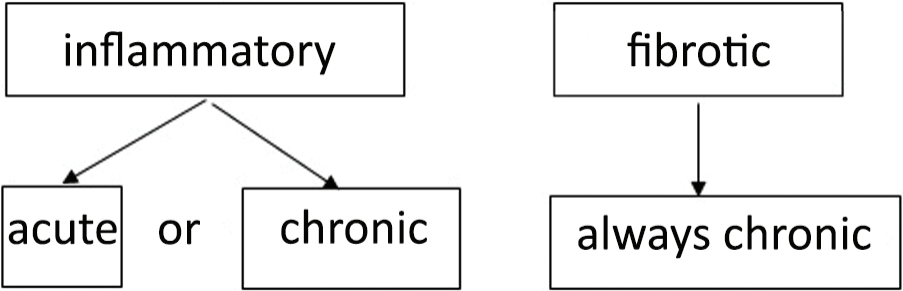
Radiologic classification of HP with possible time course according to the S2K-HP guideline (2).

A fibrotic form of HP will always be seen in a clinically chronic setting (Figure 1). In this chronic disease process, traction bronchiectasis and honeycomb cysts will never regress. Depending on various factors (allergen avoidance, therapy, individual susceptibility, etc.), progression is more likely. In contrast, the clinically acute, radiologically inflammatory phenotype may either show a restitutio ad integrum or remain stable. In fact, inflammatory signs can be morphologically detectable on HR-CT over a long period without clear signs of fibrosis appearing (Figure 1). 

## Discussion of HR-CT signs regarding reversibility and irreversibility (Table 1) 

Traction bronchiectasis and honeycomb cysts are irreversible, as are the associated irregular reticulations. These structural changes lead to a distortion of architecture. 

Air trapping is indicative of an obstructive disease of the small airways. It presents as a geographically configured mosaic pattern in which density differences increase during expiration. The pathologically hypodense (“dark”) areas correspond to overinflated pulmonary lobules. They do not decrease in volume or increase in density during expiration. Within the overinflated lobules, the vascular calibers are reduced due to hypoxic vasoconstriction (secondary mosaic perfusion). Depending on how many lobules are affected, isolated, polyhedrally configured hypodense areas or multilocular to widespread density reductions can be observed. 

Air trapping is most commonly associated with obstructive lung diseases, such as chronic obstructive pulmonary disease or asthma. However, “classical” interstitial lung diseases (ILDs) (e.g., sarcoidosis, respiratory bronchiolitis with ILD) can also be associated with air trapping. This also includes ILDs with fibrosis, which inevitably impair the airways up to the bronchioles due to architectural distortion and retraction [3, 4]. Recent studies have shown, that early stages of IPF (“early fibrosis”) already exhibit structural and functional changes in the small airways [5]. 

In contrast, HP is characterized by a mixed bronchiolar and interstitial component. Air trapping is a primary component of the disease and can be found to varying degrees in both the inflammatory and fibrotic phenotypes. 

In both phenotypes, air trapping can persist. Chung et al. [6] found that air trapping occurred more often (79%: 30/38) in HP patients without HR-CT fibrosis and less frequently in those with fibrosis (36%: 26/72), indicating a prognostic significance. In patients without fibrosis and with air trapping, it was discussed that air trapping may represent a sign of active inflammation of the airways and has the prognostic potential to suggest cases with a stable course. 

**Figure 2:**
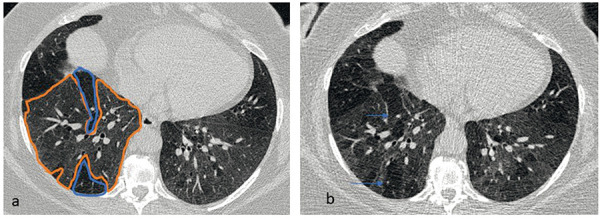
Noncontrast HR-CT obtained at inspiration in HP. Axial CT images at the level of the lower lobes. a: Initial findings; b: Low dose HR-CT after 2 years. a, b: Nearly identical manifestation of diffuse ground-glass opacities (a highlighted in orange on the right) – interrupted by hypodense (a highlighted in blue on the right) areas of air trapping. The persistence of groundglass opacities and air trapping over 2 years is consistent with fine fibrosis and chronic bronchiolitis. Note: Reduced vessel caliber in the air trapping areas due to secondary mosaic perfusion (b indicated by arrows on the right).

According to our observations, air trapping is often detectable in the chronic inflammatory phenotype on CT for a long time without radiological signs of fibrosis (Figure 2). 

Patho-histologically, air trapping in HP results from an obliterative bronchiolitis, characterized by cellular bronchiolitis with granulation tissue formation in the bronchial lumen, leading to subsequent lumen obstruction [7]. Whether a developing “scarring”, or constrictive bronchiolitis in the course leads to prolonged persistence and/or potential irreversibility has so far not been investigated, to our knowledge. 

In the fibrotic phenotype, Choe et al. [8] also described the persistence of air trapping, as the air trapping areas remained spared from the progressing fibrosis. Similarly, Chung et al. [6] noted that, in fibrotic HP, the air trapping areas were “protected” from fibrosis. In the fibrotic form, air trapping is considered a diagnostically relevant indicator of the underlying disease. The diagnostic certainty increases with the quantity of air trapping on expiratory HR-CT [9]. 

Ground-glass opacity is manifested in HR-CT as a diffuse or multilocular focal density increase without mosaic perfusion (no vascular caliber differences). The causes of ground-glass opacity are diverse (inflammatory cells or fluid in alveoli/alveolar walls or interstitially, increased capillary blood volume, etc.). 

Ground-glass opacity without accompanying fibrosis is primarily seen as a sign of inflammation in ILDs [10], suggesting potential reversibility. This is true for acute, purely inflammatory HP. Both centrilobular ground-glass nodules, which represent cellular bronchiolitis [11], and diffuse ground-glass opacities can regress in these cases (with therapy and/or allergen avoidance). 

**Figure 3:**
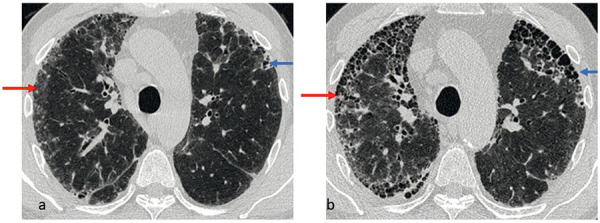
Noncontrast HRCT obtained at inspiration in fibrotic HP. Axial CT images at the level of the upper lobes. a: Initial findings; b: progression after 7 years. a: Irregular reticulations with traction bronchiolectasis subpleural, predominantly on the left (blue arrow). Subpleural on the right – next to subtle reticulation and traction bronchiolectasis – primarily with ground-glass opacity (red arrow). b: Progression of fibrosis in the previous areas of ground-glass opacities (red arrow). Retrospectively ground-glass opacities were the initial finding of fibrosis. Honeycombing in the subpleural areas of both lungs were new (see also the blue arrow in b, left upper lobe). Air trapping is not detectable in this case.

In contrast, ground-glass opacity can persist for years, independent of therapy, without clear signs of fibrosis being detectable on HR-CT. In these cases, the question arises whether the ground-glass opacity represents a fine, stationary fibrosis below the resolution capacity of HR-CT [12, 13, 14]. In such cases, irreversibility would have to be assumed (Figure 2). In fibrotic HP, chronic ground-glass opacity is only considered inflammatory if it is outside of apparent fibrosis. Within the fibrous areas, ground-glass opacity must be regarded as the initial stage of manifest fibrosis [12, 13] (Figure 3). 

The 3-density sign describes the combination of ground-glass opacity (or consolidation), air trapping, and normal parenchyma [15]. This sign indicates the combination of an infiltrative process with an obstructive process. The 3-density sign has a high specificity for the presence of HP but is not pathognomonic. The individual components regarding reversibility versus irreversibility have already been discussed. 

Cysts are more frequently described in chronic fibrotic HP [16] than in acute HP [17]. They are believed to be a consequence of bronchiolar obstruction. The more frequent occurrence in the chronic fibrotic form suggests the irreversibility of the cysts. 

## Authors’ contributions 

BR wrote the paper with input from all authors. AG especially edited the pathological aspects. BR designed the figure and table, choosed the CT-images and performed the literature research. All authors read and approved the final version of the manuscript. 

## Funding 

None. 

## Conflict of interest 

B. Rehbock has received honoraria for lectures from AstraZeneca, Boehringer Ingelheim, Chiesi, Novartis, and Sanofi-Aventis. 

A. Gschwendtner has received honoraria for lectures from AstraZeneca, Boehringer Ingelheim, and exact sciences. 

O.W. Hamer has received honoraria for lectures from Boehringer Ingelheim and Roche Pharma AG. 

**Table 1.** HR-CT signs with possible irreversibility and certain irreversibility.

**Possible irreversible signs**	**Certainly irreversible signs**
Ground-glass opacity	Irregular reticulation
3-density sign	Traction bronchiectasis
Air trapping	Honeycomb cysts
	Architectural distortion
	Cysts

## References

[1] Raghu G Remy-Jardin M Ryerson CJ Myers JL Kreuter M Vasakova M Bargagli E Chung JH Collins BF Bendstrup E Chami HA Chua AT Corte TJ Dalphin JC Danoff SK Diaz-Mendoza J Duggal A Egashira R Ewing T Gulati M Diagnosis of Hypersensitivity Pneumonitis in Adults. An Official ATS/JRS/ALAT Clinical Practice Guideline. Am J Respir Crit Care Med. 2020; 202: e36–e69. 32706311 10.1164/rccm.202005-2032ST PMC7397797

[2] Koschel D Behr J Berger M Bonella F Hamer O Joest M 2024; 78: 963–1002. 10.1055/a-2369-8458 39227017

[3] Yagihashi K Huckleberry J Colby TV Tazelaar HD Zach J Sundaram B Pipavath S Schwarz MI Lynch DA Radiologic-pathologic discordance in biopsy-proven usual interstitial pneumonia. Eur Respir J. 2016; 47: 1189–1197. 26917616 10.1183/13993003.01680-2015

[4] García-Manso IG Arenas-Jiménez J García-Sevila R Ruiz-Alcaraz S Sirera-Matilla M García-Garrigós E Martínez-García MÁ Hernández-Blasco L Author Correction: Mosaic attenuation in non-fibrotic areas as a predictor of non-usual interstitial pneumonia pathologic diagnosis. Sci Rep. 2022; 12: 9293 35661122 10.1038/s41598-022-13314-x PMC9166730

[5] Verleden SE Tanabe N McDonough JE Vasilescu DM Xu F Wuyts WA Piloni D De Sadeleer L Willems S Mai C Hostens J Cooper JD Verbeken EK Verschakelen J Galban CJ Van Raemdonck DE Colby TV Decramer M Verleden GM Kaminski N Small airways pathology in idiopathic pulmonary fibrosis: a retrospective cohort study. Lancet Respir Med. 2020; 8: 573–584. 32061334 10.1016/S2213-2600(19)30356-X PMC7292784

[6] Chung JH Zhan X Cao M Koelsch TL Gomez Manjarres DC Brown KK Presence of Air Trapping and Mosaic Attenuation on Chest Computed Tomography Predicts Survival in Chronic Hypersensitivity Pneumonitis. Annals of the American Thoracic Society. 2017; 14: 1533–1538. 28513215 10.1513/AnnalsATS.201701-035OC

[7] Syazatul SSA Piciucchi S Tomassetti S Ravaglia C Dubini A Poletti V Cryobiopsy in the diagnosis of bronchiolitis: a retrospective analysis of twenty-three consecutive patients. Scientific reports. 2020; 10: 10906. 32616807 10.1038/s41598-020-67938-y PMC7331727

[8] Choe J Chae EJ Kim YJ Do K-H Song JS Song JW Serial changes of CT findings in patients with chronic hypersensitivity pneumonitis: imaging trajectories and predictors of fibrotic progression and acute exacerbation. Eur Radiol. 2021; 31: 3993–4003. 33241510 10.1007/s00330-020-07469-2

[9] Marinescu D-C Hague CJ Muller NL Murphy D Churg A Wright JL Al-Arnawoot A Bilawich AM Bourgouin P Cox G Durand C Elliot T Ellis J Fisher JH Fladeland D Grant-Orser A Goobie GC Guenther Z Haider E Hambly N Integration and Application of Radiologic Patterns From Clinical Practice Guidelines on Idiopathic Pulmonary Fibrosis and Fibrotic Hypersensitivity Pneumonitis. Chest. 2023; 164: 1466–1475. 37541339 10.1016/j.chest.2023.07.068

[10] Remy-Jardin M Giraud F Remy J Copin MC Gosselin B Duhamel A Importance of ground-glass attenuation in chronic diffuse infiltrative lung disease: pathologic-CT correlation. Radiology. 1993; 189: 693–698. 8234692 10.1148/radiology.189.3.8234692

[11] Martínez de Alegría Alonso A Bermúdez Naveira A Uceda Navarro D Domínguez Robla M Expiratory CT scan: When to do it and how to interpret it. Radiologia (Engl Ed). 2023; 65: 352–361. 37516488 10.1016/j.rxeng.2023.01.008

[12] Müller NL Staples CA Miller RR Vedal S Thurlbeck WM Ostrow DN Disease activity in idiopathic pulmonary fibrosis: CT and pathologic correlation. Radiology. 1987; 165: 731–734. 3685351 10.1148/radiology.165.3.3685351

[13] Remy-Jardin M Remy J Artaud D Fribourg M Bonne F Copin M-C Gosselin B HRCT – Pathologic Correlations in Chronic Diffuse Infiltrative Lung Disease. In: Radiologic-Pathologic Correlations from Head to Toe. Understanding the Manifestations of Disease. Berlin, Heidelberg: Springer-Verlag Berlin Heidelberg. 2005.

[14] Devaraj A Milanese G Sverzellati N Thoracic computed tomography in the progressive fibrotic phenotype. Curr Opin Pulm Med. 2021; 27: 350–354. 34224434 10.1097/MCP.0000000000000804

[15] Chong BJ Kanne JP Chung JH Headcheese sign. J Thorac Imaging. 2014; 29: W13. 24361976 10.1097/RTI.0000000000000067

[16] Silva CIS Müller NL Lynch DA Curran-Everett D Brown KK Kyung Soo L Chronic hypersensitivity pneumonitis: differentiation from idiopathic pulmonary fibrosis and nonspecific interstitial pneumonia by using thin-section CT. Radiology. 2008; 246: 288–297. 18096541 10.1148/radiol.2453061881

[17] Franquet T Hansell DM Senbanjo T Remy-Jardin M Müller NL Lung cysts in subacute hypersensitivity pneumonitis. J Comput Assist Tomogr. 2003; 27: 475–478. 12886127 10.1097/00004728-200307000-00003

